# Suitability of Fiber Lengths for Hot Mix Asphalt with Different Nominal Maximum Aggregate Size: A Pilot Experimental Investigation

**DOI:** 10.3390/ma13173685

**Published:** 2020-08-20

**Authors:** Keke Lou, Peng Xiao, Aihong Kang, Zhengguang Wu, Pengcheng Lu

**Affiliations:** 1 College of Civil Science and Engineering, Yangzhou University, Yangzhou 225127, China; lkkyzu@163.com (K.L.); pengxiao@yzu.edu.cn (P.X.); zgwu@yzu.edu.cn (Z.W.); 006649@yzu.edu.cn (P.L.); 2 Research Center for Basalt Fiber Composite Construction Materials, Yangzhou University, Yangzhou 225127, China

**Keywords:** hot mix asphalt, basalt fiber, fiber length, mechanical performance

## Abstract

Fiber length is a key parameter for the mixture design of basalt fiber-reinforced hot mix asphalt (HMA), which significantly affects the mix performance. To evaluate the suitability of fiber lengths for HMA with different nominal maximum aggregate size (NMAS), basalt fiber with the lengths of 3, 6, 9, 12, and 15 mm were selected for dense graded gradations with different NMASs (namely, SUP-13, SUP-20, and SUP-25), so as to prepare the fiber-reinforced HMA mixtures. Then, the mix performance was evaluated by an indirect tensile asphalt cracking test (IDEAL-CT), a four-point bending beam fatigue test, a wheel tracking test, a uniaxial penetration test, a low temperature bending beam test, and a freeze-thaw splitting test. Based on the performance results, the optimum fiber length for each mix gradation was proposed by the normalization method. The results showed that adding basalt fiber can enhance the comprehensive performance of all three types of HMA to a great extent. Furthermore, fiber length presented remarkable impact on the crack resistance, the fatigue resistance of the HMA, and the low temperature crack resistance, but it had limited influence on the high temperature deformation resistance, and water stability. The optimum fiber length for SUP-13, SUP-20, and SUP-25 was 6, 9, and 12 mm, respectively.

## 1. Introduction

### 1.1. Background

Hot mix asphalt is widely used for the construction of roadways and airports all over the world because of its good riding quality, recyclability, stability, durability against various weather conditions and applied loads, and low noise [1,2]. However, owing to increases in traffic volume and the number of heavy vehicles, as well as influences from environmental factors, asphalt pavement often suffers from premature deterioration far before its design life, such as cracking (especially in winter) and rutting (especially in summer) [3], seriously affecting the pavement performance, traffic comfort, and safety [4,5,6].

Introducing fibers into the HMA has proved to be an effective way to improve the mechanical and functional performance of the asphalt mixture, and it prolongs the fatigue life of the pavement structure [7,8,9,10,11,12]. Researches on the use of fibers in HMA date back to the 1950s, when asbestos fiber was first used to enhance the performance of asphalt mixture; it was then replaced by polyester fibers because of environmental pollution and health hazards [13]. Polyacrylonitrile, lignin, brucite, glass, and steel fibers are currently commonly used as an alternative to deal with the main asphalt pavement problems [14,15,16,17,18,19,20]. The application of chopped basalt fibers into the asphalt mixture started in the 1990s and became a mainstream direction of research and application due to a higher work temperature and better mechanical performance, in addition to the superior renewability and durability [21]. Basalt fiber-reinforced asphalt pavement was first constructed in the United States, and showed performance superior to other fibers. In China, over 1000 km of asphalt pavement were paved with a basalt fiber-reinforced mixture over the past years.

Engineering practice and research showed that the reinforcing effect of the fibers on the mixture performance depends on multiple factors, for example, fiber dosages, fiber types (basalt fiber, lignin fiber, polyacrylonitrile fiber, glass fiber, etc.), fiber mechanical properties (elasticity modulus, tensile strength, etc.), asphalt properties, and mixture gradation. Many literatures were dedicated to the above factors [22,23,24,25,26], and the results indicated that some factors have to be strictly controlled to achieve the desirable mixture performance. Fortunately, the fiber properties, the proposed optimum fiber dosage selection for different mixture gradations, and the construction processes are specified in the newly issued guideline “Technical Guideline for Construction of Asphalt Pavement with Basalt Fiber” (T/CHTS 10016-2019) [27] and the provincial specification “Standard specification for construction of Asphalt Pavement with Basalt Fiber” (DB32/T 3710-2019) [28], which contribute to the standardized application of basalt fiber in the asphalt mixture and ensure the reinforcing effect to some extent.

In fact, fiber length is also a key parameter in the process of mixture design; a few researches have pointed out that fiber length can significantly affect the properties of asphalt binder and the performances of the fiber-reinforced asphalt mixture. Li adopted the mesh-basket drain down test and cone penetration test for a basalt fiber-reinforced asphalt binder with different lengths to determine the favorable fiber length, and 6 mm was recommended [16]. Tan’s research indicated that basalt fiber length has a more significant effect on Marshall stability than basalt fiber content and the asphalt-aggregate ratio [29]. Pei’s research showed that under the same content, the viscosity of aramid fiber modified asphalt will also increase with the use of longer aramid fibers, which can reflect the evident fiber length effect [30]. Qin proposed that the length of basalt fiber influences the contact area with asphalt mastic, which affects the asphalt adsorption and strength behavior [31]. Zhang obtained similar results through 3D numerical simulation [32]. It can be seen that those findings found a significant effect of fiber length on the mixture performance by binder test, limited mixture test, or simulation methods. However, very limited researches have been conducted to explore the effect of fiber length on the comprehensive performance of HMA with different NMAS.

### 1.2. Objectives and Significance

This study aims to investigate the suitability of fiber lengths for HMA with different NMAS, and proposes the corresponding optimum fiber length for each HMA. To achieve these objectives, five different lengths of basalt fiber and three dense graded gradations with typical NMAS were selected to produce fiber-reinforced HMA mixtures. Then the performance of the HMA was evaluated by the IDEAL cracking test (IDEAL-CT), four-point bending beam fatigue test, wheel tracking test, uniaxial penetration test, low temperature bending beam test and the freeze-thaw splitting test. Finally, the influence of fiber lengths on the mix performance was analyzed. The findings of this work provide a significant reference for basalt fiber selection used in HMA, and contribute to realizing the best enhancement effect caused by basalt fiber.

## 2. Materials and Mixing

### 2.1. Raw Materials

#### 2.1.1. Aggregates

**Figure 1:**
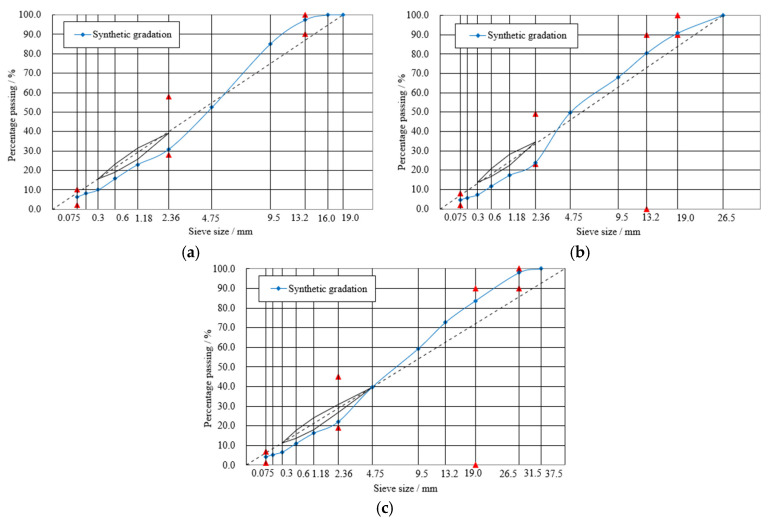
Mixture gradation curves: (**a**) SUP-13; (**b**) SUP-20; and (**c**) SUP-25.

Aggregates were obtained from the same limestone quarry located in the east of China, and the aggregate property tests were carried out based on the China standard “Test Methods of Aggregate for Highway Engineering” (JTG E42-2005) [33]. Three typical gradations commonly used for the surface, binder, and lower courses of the pavement structure were selected: the gradations with the 13.2, 19.0, and 26.5 mm nominal maximum aggregate size, named Superpave-13, Superpave-20, and Superpave-25, and abbreviated as SUP-13, SUP-20, and SUP-25, respectively. The gradation curves are shown in Figure 1.

#### 2.1.2. Asphalt Binder

A type of radial styrene-butadiene-styrene (SBS) modified asphalt with a PG grade of PG76-22 was obtained from Jiangyin Baoli (Wuxi, China). The basic physical characteristics of the asphalt binder are summarized in Table 1.

#### 2.1.3. Basalt Fiber

**Figure 2:**
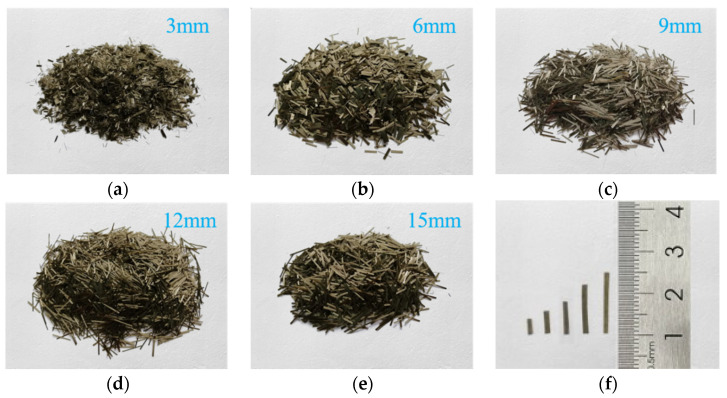
Basalt fiber, with length: (**a**) 3 mm; (**b**) 6 mm; (**c**) 9 mm; (**d**) 12 mm; (**e**) 15 mm; and (**f**) a comparison of fiber lengths.

Basalt fiber was produced by Jiangsu Tianlong (Yangzhou, China). Five different lengths of basalt fiber (3, 6, 9, 12, and 15 mm) were selected. Notice that the length is generally a multiple of 3, which is determined by the production process. The appearance is golden brown, as shown in Figure 2. Table 2 lists the properties of the basalt fiber.

### 2.2. Mix Design

The aggregate and asphalt binder sources remained constant for this study. The fiber content was also held constant at 0.3% by weight of the mixture. Mix designs were conducted following Superpave guidelines [34]. The optimum asphalt content (OAC) of each mixture was determined at 4.0% air voids. The results from the Superpave test, including OAC, voids in mineral aggregate (VMA), voids filled with asphalt (VFA), and volume of air voids (VV), are summarized in Table 3. It can be seen that for each mix type, compared with the neat mixture, OAC increased by 0.1% when basalt fiber was used, and the volumetric properties, such as VMA, VFA, and VV, remained at the same level.

### 2.3. Preparation for Fiber-Reinforced HMA

The preparation process of fiber-reinforced HMA is similar to that for conventional HMA. The only difference is that the basalt fiber should be mixed with the aggregates for 90 seconds before adding asphalt, so as to make the fiber dispersed among the aggregates. The groups of each gradation reinforced with three different single fiber lengths were fabricated, along with a neat group, as shown in Table 4. Specifically, fiber lengths of 3, 6, and 9 mm were selected for SUP-13, while lengths of 6, 9, and 12mm were selected for SUP-20, and lengths of 9, 12, and 15 mm were selected for SUP-25. The air voids of all the specimens were controlled at 7.0% ± 0.5%.

## 3. Test Methods

### 3.1. IDEAL Cracking Test

**Figure 3:**
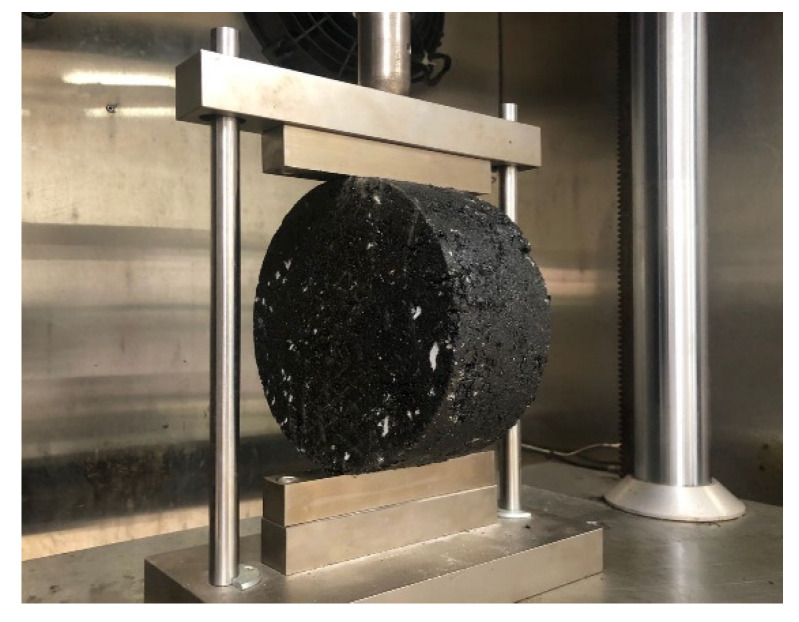
IDEAL cracking test (IDEAL-CT).

The IDEAL cracking test (IDEAL-CT) is a simple (no drilling, cutting, gluing or notching of the specimens, etc.), low variability (good reproducibility), and efficient test (test completion in less than 2 min) [35]. The dimensions of the specimens were normally 150 mm in diameter and 62 mm in thickness, as shown in Figure 3. This test was conducted at 25 °C with a loading rate of 50 mm/min.

The *CT_Index_* was used to evaluate the crack resistance, which is defined in Equation (1). *CT_Index_* is negatively correlated with the crack propagation rate of the asphalt mixture.

(1) $$CT_{Index}=\frac{G_{f}}{\left|m_{75}\right|}\times \left(\frac{l_{75}}{D}\right),$$

where $G_{f}$ is the fracture energy, j/m^2^; $\left|m_{75}\right|$ is the absolute value of the slope between the post-peak point 85 and the post-peak point 65; $l_{75}$ is the displacement at 75% peak, mm; and D is the diameter of the specimen, mm.

### 3.2. Four-Point Bending Beam Fatigue Test

**Figure 4:**
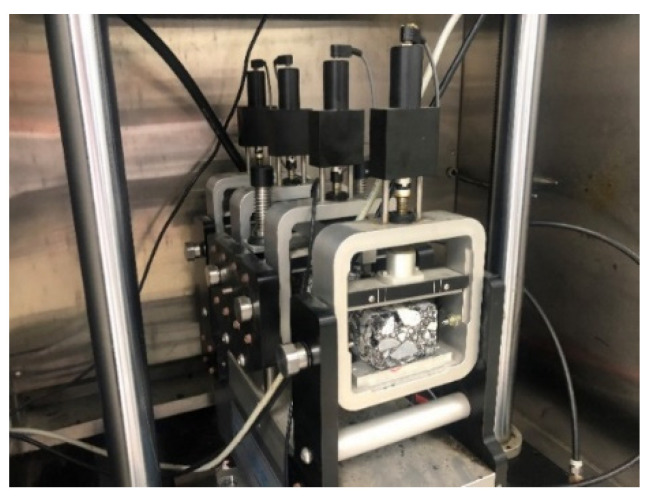
Four-point bending beam fatigue test.

The four-point bending beam fatigue test represents one of the best experimental methods for determining the fatigue life of an asphalt mixture. In this study, the test was conducted in accordance with the Chinese standard JTG E20-2011 (T0739) [36]. The test temperature was 15 °C. The size of the specimens was 300 mm × 50 mm × 50 mm, as shown in Figure 4. The strain control mode was chosen at strain levels of 450, 650, and 850 με. A sine wave was used for loading, with a frequency of 10 Hz. It is assumed that the specimen failed when the stiffness modulus decreased to 50% of its original modulus level, which is defined as the fatigue life N_f,50_. The fatigue life N_f,50_ was selected to evaluate the fatigue resistance.

### 3.3. Wheel Tracking Test

A wheel tracking test was carried out according to the Chinese standard JTG E20-2011 (T0719) [36]. The size of the specimens was 300 mm × 300 mm × 50 mm and was maintained at the temperature of 60 °C before the test. The wheel speed was 42 cycles/min with a load of 0.7 MPa. Dynamic stability (DS) was used to evaluate the high temperature deformation resistance, which is defined in Equation (2).

(2) $$DS=\frac{(t_{2}-t_{1})\times N}{d_{2}-d_{1}}\times C_{1}\times C_{2},$$

where $d_{1}$ is the deformation depth at 45 min (t_1_), mm; $d_{2}$ is the deformation depth at 60 min (t_2_), mm; $C_{1}$ and $C_{2}$ = 1.0 in this study, are experimental coefficients; and *N* means the number of wheel passings in one minute, where N= 42 cycles/min.

### 3.4. Uniaxial Penetration Test

**Figure 5:**
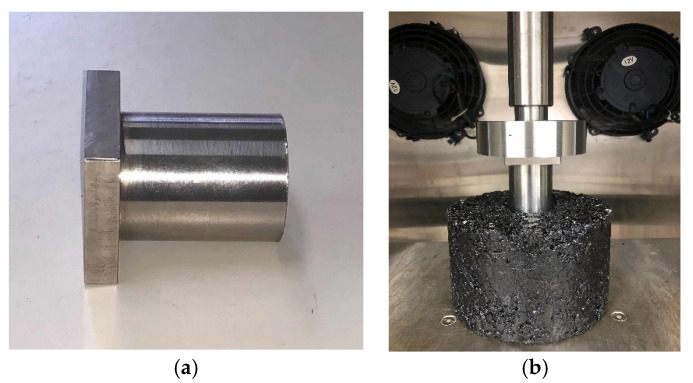
Uniaxial penetration shear test: (**a**) plunger; and (**b**) experimental setup.

The uniaxial penetration test (UPT) is a new test method adopted by the Chinese specification JTG D50-2017 [37]. This test procedure was used to determine the high-temperature shear strength of the asphalt mixture. For the UPT, a cylindrical specimen was placed inside the chamber of a universal testing machine (UTM), and the load was applied through a metal plunger. All tests were conducted in the uniaxial mode without confinement. The dimensions of the specimen were normally 150 mm in diameter and 100 mm in thickness. The dimension of the plunger was 42 mm in diameter and 50 mm in thickness, as shown in Figure 5. This test was conducted at 60 °C with a loading rate of 1 mm/min. Additional details about the mechanism and procedure of this test are available in reference [38,39].

The shear strength of the asphalt mixture can be expressed by Equations (3) and (4):

(3) $$\sigma_{p}=F/A_{c}$$

(4) $$\tau_{0}=f\times \sigma_{p},$$

where $\sigma_{p}$ is the penetration stress, MPa; F is the maximum load, N; $A_{c}$ is area of the cross-section, mm^2^; $\tau_{0}$ is the shear strength, MPa; and f is the sample dimension correction coefficient, where f = 0.350.

### 3.5. Low Temperature Bending Beam Test

A low temperature bending beam test was conducted according to the Chinese standard JTG E20-2011 (T0715) [36]. This test was run at a temperature of −10 °C. The size of the specimens was 250 × 30 × 35 mm. The loading rate was 50 mm/min. Failure strain was used to evaluate the low temperature crack resistance, which is defined in Equation (5). The failure strain is positively correlated with the low temperature crack resistance.

(5) $$\varepsilon_{B}=\frac{6h_{2}l}{L^{2}},$$

where $h_{2}$ is the height of specimen, mm; l is the deflection of mid-span at failure, mm; and L is the span of the specimen, mm.

### 3.6. Freeze-Thaw Splitting Test

A freeze-thaw splitting test was conducted in accordance with the Chinese standard JTG E20-2011 (T0729) [36]. Standard Marshall specimens were used in this study. The samples were divided into two groups. The first group was subjected to the unconditioned splitting test. The rest of the groups were subjected to the conditioned test, including vacuum soaking, freezing at −18 °C ± 2 °C for 16 h, heated up in a thermostatic tank at 60 °C for 24 h, and finally immersed into a thermostatic tank at 25 °C for 2 h before the splitting test. The split strength ratio is used to evaluate the water stability, which is defined in Equation (6):

(6) $$S_{TSR}=\frac{\bar{R_{T2}}}{\bar{R_{T1}}}\times 100,$$

where $\bar{R_{T2}}$ is the average value of the indirect tension strength of conditioned samples, MPa; and $\bar{R_{T1}}$ is the average value of the indirect tension strength of unconditioned samples, MPa.

## 4. Experimental Results and Discussions

### 4.1. Crack Resistance

**Figure 6:**
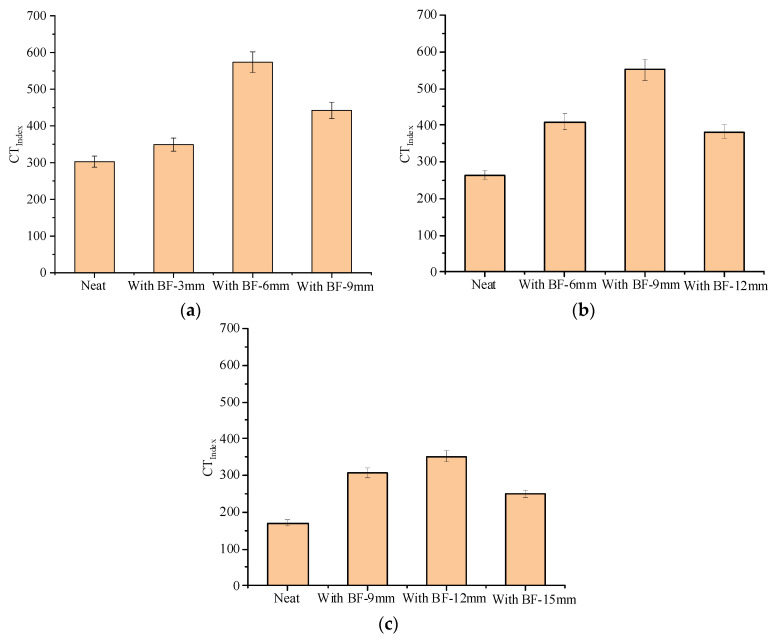
IDEAL cracking test results: (**a**) SUP-13; (**b**) SUP-20; and (**c**) SUP-25.

The IDEAL cracking test results and standard deviations are shown in Figure 6 and Table S1. It can be observed from Figure 6 that compared with the neat one, the *CT_Index_* of SUP-13 with fiber lengths of 3, 6, and 9 mm increased by 15.3%, 89.6%, and 46.2%, respectively. SUP-20 with fiber lengths of 6, 9, and 12 mm increased by 54.3%, 108.2%, and 44.4%, respectively. SUP-25 with fiber lengths of 9, 12, and 15 mm increased by 80.3%, 106.3%, and 46.5%, respectively. The coefficient of variation is within 6%. This indicates that basalt fiber can significantly enhance the crack resistance of the asphalt mixture. The explanation could be that basalt fiber works as a “bridge”, so as to effectively prevent the crack initiation and propagation process in the asphalt mixture, which is consistent with the results from reference [3]. Furthermore, it is noticeable that the *CT_Index_* of asphalt mixture is very sensitive to the variation of fiber length. At the same dosage, fiber with the length of 6 mm has the best improvement effect on SUP-13, followed by the fiber lengths of 9 and 3 mm. The length of 9 mm has the best improvement effect on SUP-20, followed by lengths of 6 and 12 mm. Fiber with the length of 12 mm has the best improvement effect on SUP-25, followed by 9 and 15 mm. The optimum fiber length is closely related to the nominal maximum particle size of the asphalt mixture, and larger nominal particle sizes require longer fibers. The results may be due to the differences in the space formed by the intercalation between coarse aggregates.

### 4.2. Fatigue Resistance

**Figure 7:**
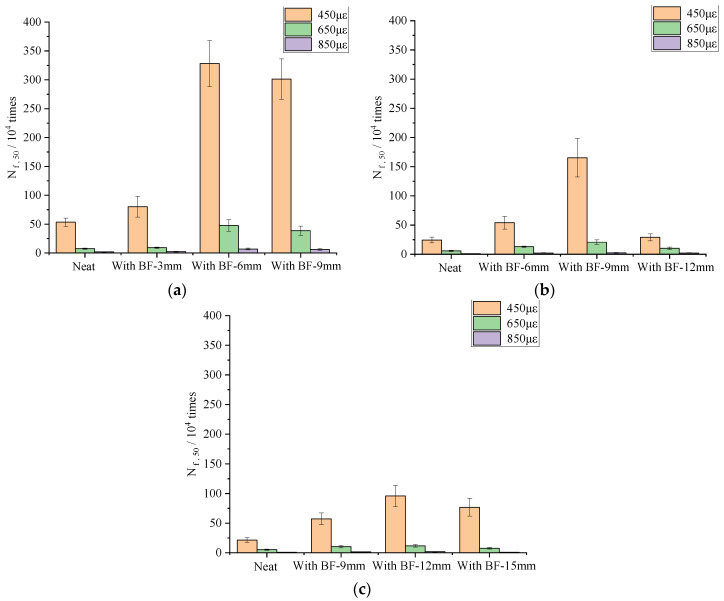
Four-point bending beam fatigue test results: (**a**) SUP-13; (**b**) SUP-20; and (**c**) SUP-25.

Figure 7 and Table S2 illustrate the results and standard deviation from the four-point bending beam fatigue test. As shown in Figure 7, compared with the neat one, the fatigue life N_f,50_ of the asphalt mixture with basalt fiber increased dramatically at the strain level of 450 με. The fatigue life N_f,50_ of SUP-13 with fiber lengths of 3, 6, and 9 mm increased by 50.4%, 514.2%, 463.6%, respectively. The fatigue life N_f,50_ of SUP-20 with fiber length 6, 9, and 12 mm increased by 121.6%, 579.0%, 19.2%, respectively. The fatigue life N_f,50_ of SUP-25 with fiber lengths of 9, 12, and 15 mm increased by 168.5%, 349.6%, 260.3%, respectively. The coefficient of variation is within a reasonable range. Consistent upward trends of fatigue life could also be observed at the strain levels of 650 and 850 με. This visibly indicates that basalt fiber can significantly enhance the fatigue resistance of an asphalt mixture. Meanwhile, it is noticeable that the length of fiber did result in a differentiated effect. For instance, at the same dosage, fiber with the length of 6 mm has the best improvement effect on SUP-13, followed by 9 and 3 mm. Fiber with the length of 9 mm has the best improvement effect on SUP-20, followed by 6 and 12 mm. Fiber with the length of 12 mm has the best improvement effect on SUP-25 asphalt mixture, followed by 15 and 9 mm. It is obvious that the fatigue life of an asphalt mixture is very sensitive to the variation of fiber length. Similarly, the optimum fiber length is the same for the crack resistance test; in terms of fatigue resistance, 6, 9, and 12 mm are more suitable for SUP-13, SUP-20 and SUP-25, respectively. This further demonstrates that the optimum fiber length is closely related to the space between aggregates, which is affected by the mixture gradation.

### 4.3. High Temperature Deformation Resistance

**Figure 8:**
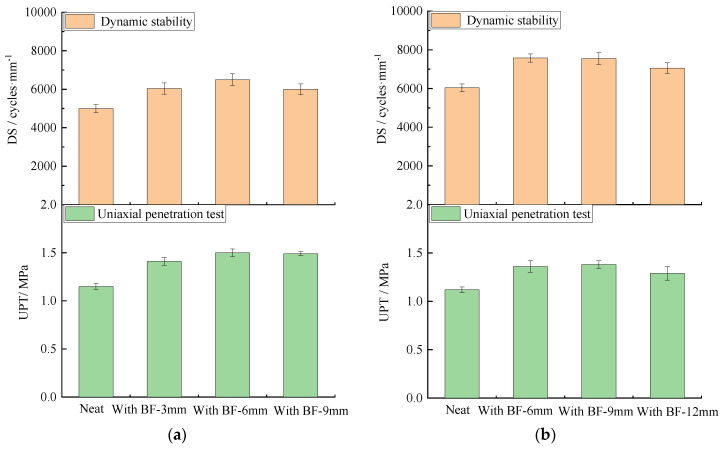

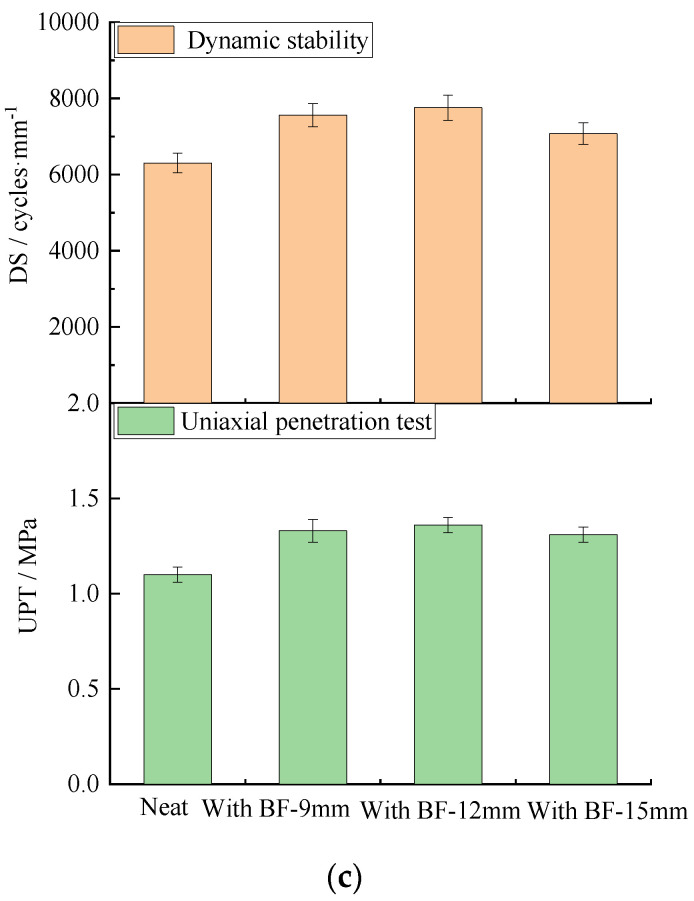
Wheel tracking and uniaxial penetration test (UPT) results: (**a**) SUP-13; (**b**) SUP-20; and (**c**) SUP-25.

The results obtained from the wheel tracking test and the uniaxial penetration test are illustrated in Figure 8 and Table S3. As shown in Figure 8, compared with the neat one, the DS of SUP-13 with fiber lengths of 3, 6, and 9 mm increased by 20.7%, 29.9%, 19.9%, respectively and the shear strength increased by 22.6%, 30.4%, and 29.6%, respectively. The DS of SUP-20 with fiber lengths of 6, 9, and 12 mm increased by 25.5%, 25.1%, 16.9%, respectively, and the shear strength increased by 21.4%, 23.2%, and 15.2%, respectively. The DS of SUP-25 with fiber lengths of 9, 12, and 15 mm increased by 20.0%, 23.1%, and 12.2%, respectively, and the shear strength increased by 20.9%, 23.6%, and 19.1%, respectively. The coefficient of variation is within a reasonable range. This indicates that the addition of basalt fibers can effectively improve the high temperature deformation resistance, no matter which length of fiber was used. Furthermore, fiber with the length of 6 mm has the best reinforcing effect for SUP-13, the length of 6 and 9 mm has a similar reinforcing effect for SUP-20, and 9 and 12 mm has a similar reinforcing effect for SUP-25. In general, the high temperature deformation resistance of HMA is not very sensitive to the variation of fiber length compared with the crack resistance and fatigue resistance for all three types of HMA with different nominal maximum aggregate size. The explanation could be that the high surface area of basalt fibers increases the proportion of structural asphalt, which improves the viscosity of asphalt, resulting in improvements of the high temperature performance of the asphalt mixture; this is consistent with the results from reference [16]. However, the surface area of basalt fiber remains at the same level as the total dosage, and the diameter was still the same, though different fiber lengths were used. Therefore, fiber length has no distinct effect on the high temperature performance of HMA.

### 4.4. Low Temperature Crack Resistance

**Figure 9:**
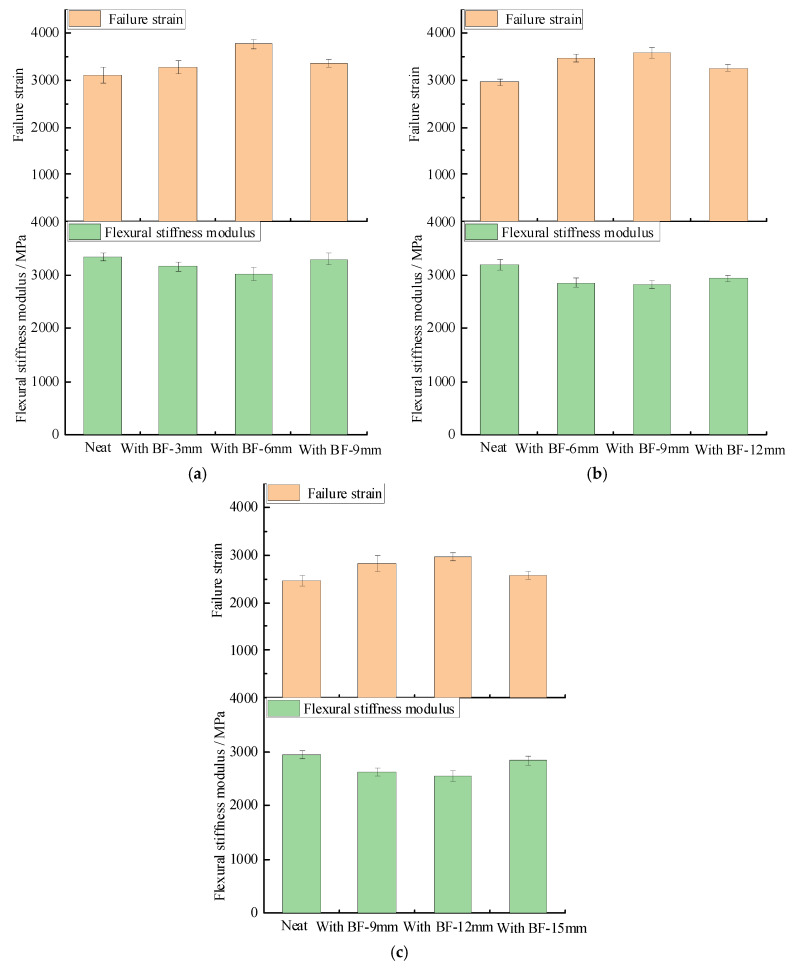
Low temperature bending beam test results: (**a**) SUP-13; (**b**) SUP-20; and (**c**) SUP-25.

Low temperature bending beam test results are shown in Figure 9 and Table S4. From Figure 9, it can be seen that compared with the neat one, the failure strain of SUP-13 with fiber lengths of 3, 6, and 9 mm increased by 5.6%, 21.2%, and 8.2%, respectively. The failure strain of SUP-20 with fiber lengths of 6, 9, and 12 mm increased by 17.2%, 21.0%, and 10.2%, respectively. The failure strain of SUP-25 with fiber lengths of 9, 12, and 15 mm increased by 14.9%, 20.0%, and 4.7%, respectively. The flexural stiffness modulus of SUP-13 with fiber lengths of 3, 6, and 9 mm decreased by 5.5%, 9.8%, and 1.1%, respectively. The flexural stiffness modulus of SUP-20 with fiber lengths of 6, 9, and 12 mm decreased by 10.7%, 11.7%, and 8.1%, respectively. The flexural stiffness modulus of SUP-25 with fiber lengths of 9, 12, and 15 mm decreased by 10.9%, 13.6%, and 3.9%, respectively. The coefficient of variation is within a reasonable range. It means that basalt fiber can effectively improve the low temperature crack resistance of the asphalt mixture. The explanation could be that basalt fiber works as a “bridge”, so as to effectively prevent the crack initiation and propagation process in the asphalt mixture, which leads to the increase of failure strain and a decrease of the flexural stiffness modulus, which is consistent with the results from reference [24]. Furthermore, for the aspect of failure strain, the variation range of SUP-13 is 5.6–21.2% with fiber lengths of 3, 6, and 9 mm. The variation range of SUP-20 is 10.2–21.0% with fiber lengths of 6, 9, and 12 mm. The variation range of SUP-25 is 4.7–20.0% with fiber lengths of 9, 12, and 15 mm. It can be seen that the variation of fiber lengths also has a significant influence on the low temperature crack resistance; this could be due to the fiber length affecting the embedding depth at the fracture of the mixture.

### 4.5. Water Stability

**Figure 10:**
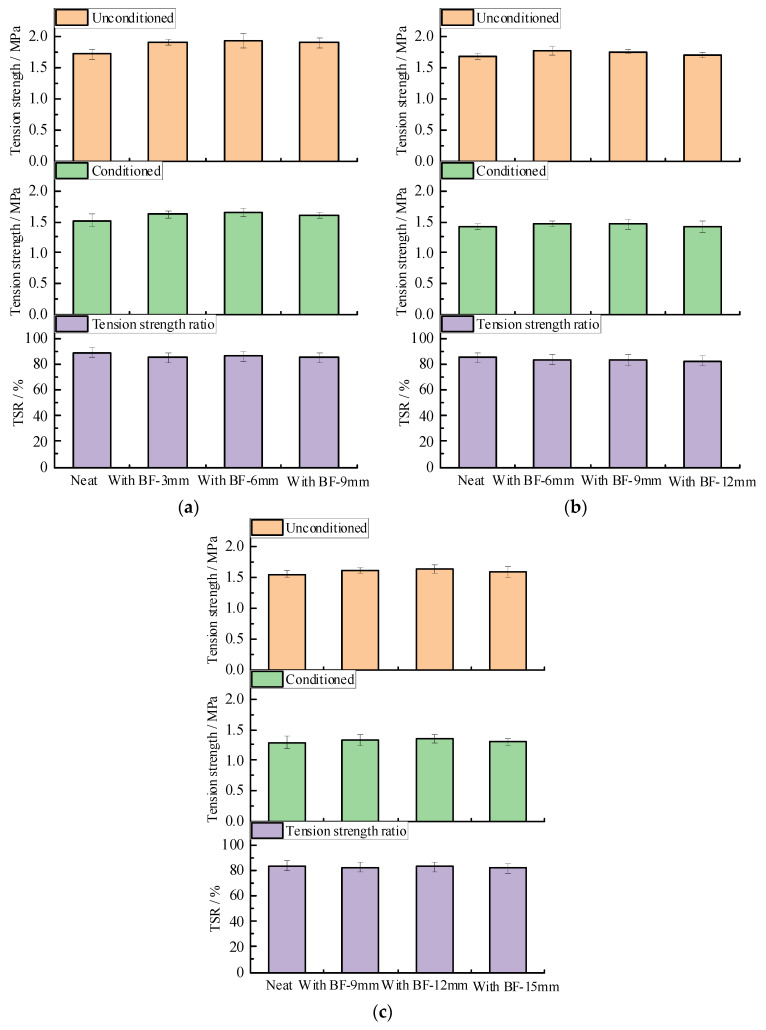
Freeze-thaw splitting test results: (**a**) SUP-13; (**b**) SUP-20; and (**c**) SUP-25.

The freeze-thaw splitting test results and standard deviation are shown in Figure 10 and Table S5. It can be observed from Figure 10 that compared with the neat mixture, both the unconditioned and conditioned indirect tension strengths of the asphalt mixture increase slightly after adding basalt fibers, regardless of the fiber length; this indicates that the fiber-reinforced samples possess higher strength after the harsh freeze-thaw procedures. The indirect tension strength ratios tend to decrease slightly. The standard deviation is within a reasonable range. It seems that the asphalt mixture with basalt fiber possesses comparable water stability as that of the neat one, and the water stability is also not sensitive to the variation of fiber length.

### 4.6. Comprehensive Analysis

It can be observed from Section 4.1, Section 4.2, Section 4.3, Section 4.4 and Section 4.5 that fiber length affects the mix performance measures: crack resistance, fatigue resistance, high temperature deformation resistance, low temperature crack resistance, and water stability, to various degrees. In order to more clearly evaluate the suitability of fiber lengths for dense graded gradation with different nominal maximum aggregate size, a normalization method was adopted in this study. Each performance of the neat mixture is considered as the benchmark, which is marked as “1”. Then, for each performance, the ratio of measured values to those of the neat mixture was calculated for every gradation. Finally, the normalized values of the mixture with each fiber length were summed, so as to represent the enhancement effect caused by different fiber lengths, as shown in Table 5.

It can be observed from Table 5 that compared with the neat mixture, adding basalt fiber can enhance the performance of HMA to some extent. The normalized results of crack resistance, fatigue resistance, and low temperature crack resistance are in the range of 1.15–2.08, 1.19–6.79, and 1.06–1.21, respectively. Therefore, fiber length has a great influence on crack resistance, fatigue resistance, and the low temperature performance of HMA. However, the normalized results of high temperature deformation resistance and water stability are in the range of 1.16–1.30 and 1.02–1.09, respectively, indicating that fiber length has limited influence on the high temperature deformation resistance and water stability.

**Figure 11:**
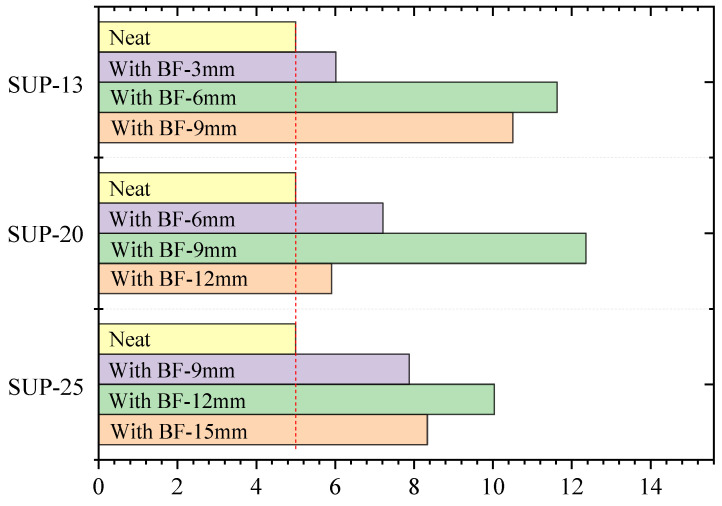
The sum of normalized results.

Figure 11 illustrates the sum values of normalized results of HMA with different fiber lengths. From Figure 11, it can be seen that, in terms of the comprehensive performance of HMA, a fiber length of 6 mm presents the best enhancement capability for SUP-13, followed by lengths of 9 and 3 mm. A fiber length of 9 mm presents the best enhancement capability for SUP-20, followed by lengths of 6 and 12 mm. A fiber length of 12 mm presents the best enhancement capability for SUP-25, followed by lengths of 15 and 9 mm. The findings indicate that fiber length suitability should be considered for HMA with different NMAS.

## 5. Conclusions

The primary objective of this work was to assess the impact of fiber lengths on the performance of basalt fiber-reinforced hot mix asphalt, and evaluate the suitability of fiber lengths for HMA with different NMAS. Basalt fiber with the lengths of 3, 6, 9, 12 and 15 mm were selected, and the comprehensive performance of fiber-reinforced HMA (SUP-13, SUP-20, SUP-25) were analyzed; the following conclusions can be derived:

- Adding basalt fiber, regardless of the length, can enhance the comprehensive performance of the hot asphalt mixture, to some extent.
- Fiber length presents significant impact on the enhancement effect on crack resistance, fatigue resistance, and low temperature crack resistance of basalt fiber-reinforced HMA with different NMAS, with the resistances increased by 15.3–108.2%, 19.2–579%, and 4.7–21.2%, respectively.
- Fiber length shows no obvious influence on the changes of high temperature deformation resistance and water stability of the selected HMA.
- Optimum fiber length is closely related to the NMAS of HMA, and larger NMAS requires longer fibers. The optimum fiber length is 6, 9, and 12 mm for SUP-13, SUP-20 and SUP-25, respectively.

The study just focused on the pilot experimental analysis. An extensive range of laboratory tests were conducted to evaluate the impact of fiber lengths on the performance of basalt fiber-reinforced hot mix asphalt. The reinforcement mechanism caused by fiber length could be considered in the future. In addition, more microscopic methods could be used to analyze the relationship between the microstructure of the asphalt mixture and the fiber length.

## Figures and Tables

**Table 1:** Basic physical characteristics of the asphalt.

Characteristics		Requirements	Results	Standard
Penetration (25 °C)/0.1 mm		60–80	71	T0604–2011
Softening point/°C		≮55	64	T0604–2011
Ductility (5cm/min, 5 °C)/cm		≮30	48	T0606–2011
Penetration Index		−0.4–1.0	0.5	T0605–2011
Vscosity (135 °C)/Pa.s		≯3	1.8	T0601–2011
Recovery of elasticity (25 °C)/%		≮65	76	T0662–2011
Softening point variation/°C		≯2.5	1.4	T0664–2011
RTFOT residue	Weight change/%	≯ ± 1.0	−0.08	T0610–2011
	Penetration ratio/%	≮60	86	T0604–2011
	Residual ductility (15 °C)/cm	≮20	37	T0605–2011

**Table 2:** Properties of the basalt fiber.

Characteristics	Requirements	Results	Standard
Diameter/μm	/	16	/
Elongation at break/%	≤3.1	2.71	GB/T 20310
Tensile strength/MPa	≥2000	2200–2500	GB/T 20310
Heat resistance, Retention rate of fracture strength/%	≥85	93	GB/T 7690.3
Acidity coefficient	≥4.5	5.2	GB/T 1549

**Table 3:** Optimum asphalt content (OAC) and volumetric properties.

Types of Mixtures	OAC/%	VMA/%	VFA/%	VV/%
SUP-13	4.7	14.09	71.33	4.04
SUP-13 + BF-3 mm	4.8	14.13	71.12	4.08
SUP-13 + BF-6 mm	4.8	14.08	71.09	4.07
SUP-13 + BF-9 mm	4.8	14.15	71.38	4.05
SUP-20	4.3	13.38	69.58	4.07
SUP-20 + BF-6 mm	4.4	13.24	69.41	4.05
SUP-20 + BF-9 mm	4.4	13.17	69.48	4.02
SUP-20 + BF-12 mm	4.4	13.26	69.68	4.02
SUP-25	4.1	12.05	66.39	4.05
SUP-25 + BF-9 mm	4.2	12.23	67.05	4.03
SUP-25 + BF-12 mm	4.2	12.14	66.64	4.05
SUP-25 + BF-15 mm	4.2	12.27	66.67	4.09

(VMA = voids in mineral aggregate; VFA = voids filled with asphalt; VV = volume of air voids).

**Table 4:** Labeling of the asphalt mixture with a single length of basalt fiber.

Types of Gradations	No.	3 mm	6 mm	9 mm	12 mm	15 mm	Dosages
Superpave-13	1-0						0
	1-1	√					0.3%
	1-2		√				0.3%
	1-3			√			0.3%
Superpave-20	2-0						0
	2-1		√				0.3%
	2-2			√			0.3%
	2-3				√		0.3%
Superpave-25	3-0						0
	3-1			√			0.3%
	3-2				√		0.3%
	3-3					√	0.3%

**Table 5:** The results of the normalization method.

Types of Gradations	No.	Crack Resistance	Fatigue Resistance	High Temperature Deformation Resistance	Low Temperature Crack Resistance	Water Stability	Sum
SUP-13	1-0	1	1	1	1	1	5
	1-1	1.15	1.50	1.22	1.06	1.09	6.02
	1-2	1.89	6.14	1.30	1.21	1.09	11.63
	1-3	1.46	5.64	1.25	1.08	1.08	10.51
SUP-20	2-0	1	1	1	1	1	5
	2-1	1.54	2.22	1.23	1.17	1.05	7.21
	2-2	2.08	6.79	1.24	1.21	1.04	12.36
	2-3	1.44	1.19	1.16	1.10	1.02	5.91
SUP-25	3-0	1	1	1	1	1	5
	3-1	1.80	2.69	1.20	1.15	1.04	7.88
	3-2	2.06	4.50	1.23	1.20	1.05	10.04
	3-3	1.46	3.60	1.16	1.10	1.02	8.34

## Supplementary Materials

The following are available online at https://www.mdpi.com/1996-1944/13/17/3685/s1, Table S1: IDEAL cracking test results; Table S2: Four-point bending beam fatigue test results; Table S3: Wheel tracking and uniaxial penetration test results; Table S4: Low temperature bending beam test results; Table S5: Freeze-thaw splitting test results.
