# Supplementary material for: Suitability of Fiber Lengths for Hot Mix Asphalt with Different Nominal Maximum Aggregate Size: A Pilot Experimental Investigation
Source: Materials (Basel). 2020 Aug 20;13(17):3685. doi: 10.3390/ma13173685 (PMC7503391; doi:10.3390/ma13173685)
Supplement: Supplementary file 1 [file materials-13-03685-s001.pdf]

materials

MDPI

Table S1. IDEAL cracking test results.

Types of gradations	CT_{Index}	Standard deviation	No.
SUP-13	302.65	15.09	Figure 6 (a)
SUP-13+BF-3mm	349.03	18.26	
SUP-13+BF-6mm	573.86	27.55	
SUP-13+BF-9mm	442.57	22.41	
SUP-20	264.85	12.03	Figure 6 (b)
SUP-20+BF-6mm	408.74	21.47	
SUP-20+BF-9mm	551.4	28.25	
SUP-20+BF-12mm	382.47	17.89	
SUP-25	170.55	9.44	Figure 6 (c)
SUP-25+BF-9mm	307.57	13.37	
SUP-25+BF-12mm	351.79	16.20	
SUP-25+BF-15mm	249.81	11.31	

Table S2. Four-point bending beam fatigue test results.

Types of gradations	$N_{f,50}$ (450με)	Standard deviation	$N_{f,50}$ (650με)	Standard deviation	$N_{f,50}$ (850με)	Standard deviation	No.
SUP-13	53.4271	7.1345	7.5341	1.4882	1.8751	0.4021	Figure 7 (a)
SUP-13+BF-3mm	80.3218	18.4651	9.2571	1.2225	2.4654	0.6147	
SUP-13+BF-6mm	328.1254	40.1315	47.5324	10.0302	6.9352	1.5412	
SUP-13+BF-9mm	301.1372	35.0275	38.5376	8.1054	6.1753	1.3018	
SUP-20	24.3572	5.1892	5.7825	1.4521	1.1237	0.3130	Figure 7 (b)
SUP-20+BF-6mm	53.9861	11.4756	12.8768	1.2489	2.2032	0.608	
SUP-20+BF-9mm	165.3753	33.1030	20.7214	4.4417	2.3501	0.6994	
SUP-20+BF-12mm	29.0357	6.1846	10.3546	2.3742	2.0872	0.4891	
SUP-25	21.3215	4.7102	5.2363	1.3658	0.7745	0.2315	Figure 7 (c)
SUP-25+BF-9mm	57.2513	10.1053	10.4268	2.1469	1.5423	0.3921	
SUP-25+BF-12mm	95.8603	18.0048	11.7834	2.1894	1.7435	0.5075	
SUP-25+BF-15mm	76.8247	14.8957	7.5672	1.5273	0.9542	0.2721	

Table S3. Wheel tracking and uniaxial penetration test results.

**Types of gradations**	**Dynamic stability**	**Standard deviation**	**Shear strength**	**Standard deviation**	**No.**
SUP-13	5001	207	1.15	0.03	**Figure 8 (a)**
SUP-13+BF-3mm	6037	292	1.41	0.04	
SUP-13+BF-6mm	6498	308	1.5	0.04	
SUP-13+BF-9mm	5995	280	1.49	0.02	
SUP-20	6036	200	1.12	0.03	**Figure 8 (b)**
SUP-20+BF-6mm	7576	213	1.36	0.06	
SUP-20+BF-9mm	7551	310	1.38	0.04	
SUP-20+BF-12mm	7054	281	1.29	0.07	
SUP-25	6300	257	1.1	0.04	**Figure 8 (c)**
SUP-25+BF-9mm	7561	309	1.33	0.06	
SUP-25+BF-12mm	7757	331	1.36	0.04	
SUP-25+BF-15mm	7072	284	1.31	0.04	

Table S4. Low temperature bending beam test results.

**Types of gradations**	**Failure strain**	**Standard deviation**	**Flexural stiffness modulus**	**Standard deviation**	**No.**
SUP-13	3100	155	3339	85	**Figure 9 (a)**
SUP-13+BF-3mm	3274	139	3156	80	
SUP-13+BF-6mm	3757	101	3011	121	
SUP-13+BF-9mm	3354	73	3302	104	
SUP-20	2953	70	3193	89	**Figure 9 (b)**
SUP-20+BF-6mm	3462	89	2851	83	
SUP-20+BF-9mm	3572	105	2819	78	
SUP-20+BF-12mm	3254	80	2935	70	
SUP-25	2463	102	2948	81	**Figure 9 (c)**
SUP-25+BF-9mm	2831	165	2628	72	
SUP-25+BF-12mm	2956	90	2547	101	
SUP-25+BF-15mm	2579	88	2834	78	

Table S5. Freeze-thaw splitting test results.

Types of gradations	indirect tension strength of unconditioned	Standard deviation	indirect tension strength of conditioned	Standard deviation	No.
SUP-13	1.71	0.08	1.52	0.10	Figure 10 (a)
SUP-13+BF-3mm	1.9	0.04	1.62	0.06	
SUP-13+BF-6mm	1.93	0.11	1.66	0.07	
SUP-13+BF-9mm	1.89	0.07	1.61	0.04	
SUP-20	1.67	0.05	1.42	0.04	Figure 10 (b)
SUP-20+BF-6mm	1.76	0.07	1.47	0.05	
SUP-20+BF-9mm	1.75	0.04	1.46	0.08	
SUP-20+BF-12mm	1.7	0.04	1.43	0.09	
SUP-25	1.54	0.06	1.29	0.11	Figure 10 (c)
SUP-25+BF-9mm	1.61	0.05	1.33	0.09	
SUP-25+BF-12mm	1.63	0.07	1.35	0.07	
SUP-25+BF-15mm	1.59	0.09	1.3	0.06	

© 2020 by the author. Licensee MDPI, Basel, Switzerland. This article is an open access article distributed under the terms and conditions of the Creative Commons Attribution (CC BY) license (http://creativecommons.org/licenses/by/4.0/).
